# Protein Hydrolysates from Anchovy (*Engraulis encrasicolus)* Waste: In Vitro and In Vivo Biological Activities

**DOI:** 10.3390/md18020086

**Published:** 2020-01-28

**Authors:** Alessia Giannetto, Emanuela Esposito, Marika Lanza, Sabrina Oliva, Kristian Riolo, Simona Di Pietro, Jessica Maria Abbate, Giovanni Briguglio, Giovanni Cassata, Luca Cicero, Francesco Macrì

**Affiliations:** 1 Department of Chemical, Biological, Pharmaceutical and Environmental Sciences. University of Messina, Viale Ferdinando Stagno d’Alcontres, 31, 98166 Messina, Italy; 2 Department of Veterinary Sciences, University of Messina, Polo Universitario SS. Annunziata, 98168 Messina, Italy; 3 Veterinary Practitian, 98167 Messina, Italy; 4 Zooprophylactic Institute of Sicily “A. Mirri”, 90129 Palermo, Italy

**Keywords:** marine organisms, anchovy *Engraulis encrasicolus*, bioactive peptides, antioxidant, anti-inflammatory

## Abstract

Fish waste utilization to obtain protein hydrolysates has been demonstrated to be a useful strategy to face both environmental and economic impacts while obtaining high-value products with remarkable biological and nutritional properties. In the present study, protein hydrolysates obtained from anchovy *Engraulis encrasicolus* (APH) by-products were assessed for their potential biological activities in both in vitro and in vivo models. The treatment with APH exerted a significant protection against LPS-induced inflammation in RAW 264.7 cells, decreasing the protein expression of pro-inflammatory mediators (i.e., COX-2) and inhibiting the nuclear translocation of NF-κB through IκB-α. Moreover, APH modulated the expression of iNOS, MnSOD and HO-1, thus decreasing the severity of oxidative stress. The supplementation of APH in the diet of ApoE knockout mice down-regulated the proinflammatory cytokines (i.e., *TNF-α*, *IL-1α*, *IL-1β*, *IL-6*) in both aorta and heart tissues, and modulated the expression of oxidative stress-related genes (*Cu/ZnSod*, *MnSod*, *Cat*, *Gpx* and *Ho*), indicating that APH can exert a beneficial role, having anti-inflammatory and antioxidant activities. The nutritional properties of APH, together with their biological activities herein reported, highlight the possibility of obtaining bioactive molecules from fish waste and encourage their use as potential nutraceuticals in food and pharmaceutical industries in the next future.

## 1. Introduction

Worldwide, food waste represents one of the main concerns because of the adverse environmental and economic consequences arising from its current strategies of management. At present, the EU waste management policy has been mainly focusing on waste prevention and search for solutions to their valorization in a circular economy context [1]. In many countries, the fish industry is a major economic source, but the inadequate management of fish processing waste is one of the priorities that the European Commission has to face. Nowadays, fishery policy aims to eliminate the discards produced from the fish manufacturing process, including the head, skin, fins and viscera, which account for more than 60% of the total biomass [2]. These discards of fish by-products represent an environmental and economic concern, and searching for strategies to convert this row materials into value-added products has been proposed. In particular, fish wastes contain up to 10–20% [3] of total fish proteins and different technologies have been developed to recover proteins and peptides from these fish by-products. Protein hydrolysis process that converts fish protein into smaller peptides, which normally contain 2–20 amino acids, has been demonstrated as an efficient way to add value to fish waste protein [4]. The so-called protein hydrolysates obtained from this process, contrary to non-hydrolyzed fish proteins, are characterized by highly balanced amino acid composition, as well as the presence of biological and nutritional properties such as antioxidant, antimicrobial, antihypertensive, anti-inflammatory, or antihyperglycemic [5] that improve their commercial interest. These properties are due to a better accessibility to the functional peptide sequences [6,7]. In particular, enzymatic hydrolysis by proteolytic enzymes has been demonstrated to be an efficient process for obtaining high-added-value products with conserved nutritional value of the source protein and a high content of essential amino acids. Noteworthy, this process is able to generate peptides characterized by different bio-activities depending on the molecular size and composition with potential use as functional food ingredients [8,9]. In literature from the last decade, it has been reported that amino acid-specific properties are shown for some amino acids with hydrophobic residues, as well as acidic (glutamic acid and aspartic acid) and basic (arginine, lysine and histidine) amino acids, meaning that they are able to act as antioxidant by acting as proton or electron donors, lipid radical scavengers [10,11,12,13] and/or metal chelators [14]. Protein hydrolysates obtained from fish by-products have recently been demonstrated to exert beneficial bioactivities in a wide range of diseases. Several authors have shown potential application of protein hydrolysates from Salmon frame and Tilapia skin gelatin as effective antidiabetic drugs in the hyperglycemic disorder [15,16]; salmon protein hydrolysates have been showed to play also an anti-inflammatory role through the inhibition of nitric oxide production and proinflammatory cytokines [17]. Despite the potential economic advantages of converting fish by-products into valuable protein hydroysates and their reported bioactivities, studies focusing on the specific biological activity of fish protein hydrolysates in animal models are still scarce. Parolini et al. [18] showed that introducing salmon protein hydrolysates in the apoE^−/−^ mouse diet can exert a lipid-independent anti-atherosclerotic activity. Effects of anchovy (*Coilia mystus*) protein hydrolysates have been reported to have a role in combating memory impairment in mice [19]. Recently, Mangano et al. [4] obtained protein hydrolysates from the European anchovy (*Engraulis encrasicolus*) and described their chemical amino acid and elemental composition. The European anchovy is a high commercial value species with a widespread distribution and represents the most important pelagic fish resource in the Mediterranean Sea [20] (http://www.iucnredlist.org/details/summary/198568/1), with high market demand. In a FAO report (2010–2014), *E. encrasicolus* was the main fished species by weight, corresponding to 25–35% of the total catches of marine fishes in Italy (http://www.fao.org/fishery/topic/16140/en). Its manufacturing processes inevitably generates waste products that represent a negative value in terms of both economic and environmental impact. To convert this series of materials into value-added products, Mangano et al. [4] applied enzymatic protein hydrolysis on anchovy *E. encrasicolus* waste, showing that the obtained protein hydrolysates displayed a remarkable presence of both essential amino acids together with key amino acids known to exert beneficial effects on human health. However, the nutritional value of the hydrolysates obtained from *E. encrasicolu* waste should be supported by animal testing with the aim of using them as ingredients for functional food production, or also as stand-alone items. More studies are needed to lead a better understanding of the mechanism through which *E. encrasicolus* protein hydrolysates exert their biological activities. This study is aimed at evaluating the anti-oxidant and anti-inflammatory potential of protein hydrolysates from anchovy *E. encrasicolus* by-products in an in vitro model of inflammation and oxidative stress, using a murine macrophage cell line (RAW 264.7) and in an in vivo model using apolipoprotein E-knockout (C57BL/6) mice. Results from this study could contribute to evaluating the feasibility of anchovy wastes in producing biologically active and health-related peptides that could meet the increasing demand for naturally bioactive food components with possible application in the prevention or treatment of human diseases in the near future. 

## 2. Results

### 2.1. In Vitro Evaluation of APH on Viability of Murine Macrophages RAW 264.7

**Figure 1:**
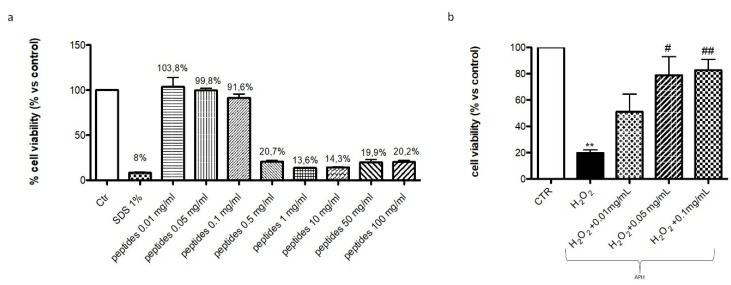
Effect of APH on cell viability of murine macrophages RAW 264.7. Cell viability was assessed 24 h after incubation with increasing concentrations (0.01–100 mg/mL) of APH, showing that only 0.01, 0.05 and 0.1 mg/mL lacked cytotoxicity (**a**). APH pre-treatment protected Raw 264.7 cells by H_2_O_2_ damage (**b**). Data are representative of at least three independent experiments. # *p* < 0.05; ## *p* < 0.01 vs. H_2_O_2_ ** *p* < 0.01 vs. Ctr.

To evaluate the effect of hydrolysates on cell viability, RAW 264.7 cells were incubated with increasing concentrations of APH: 0.01–0.05–0.1–0.5–1–10–50–100 mg/mL. After 24 h of incubation, hydrolysates at the concentration of 0.01, 0.05, 0.1 mg/mL showed a viability almost comparable to control group (95%) while the higher concentrations were inxic (20%) (Figure 1). 

### 2.2. In Vitro Effect of APH on Inflammatory Pathway of Macrophages RAW 264.7

**Figure 2:**
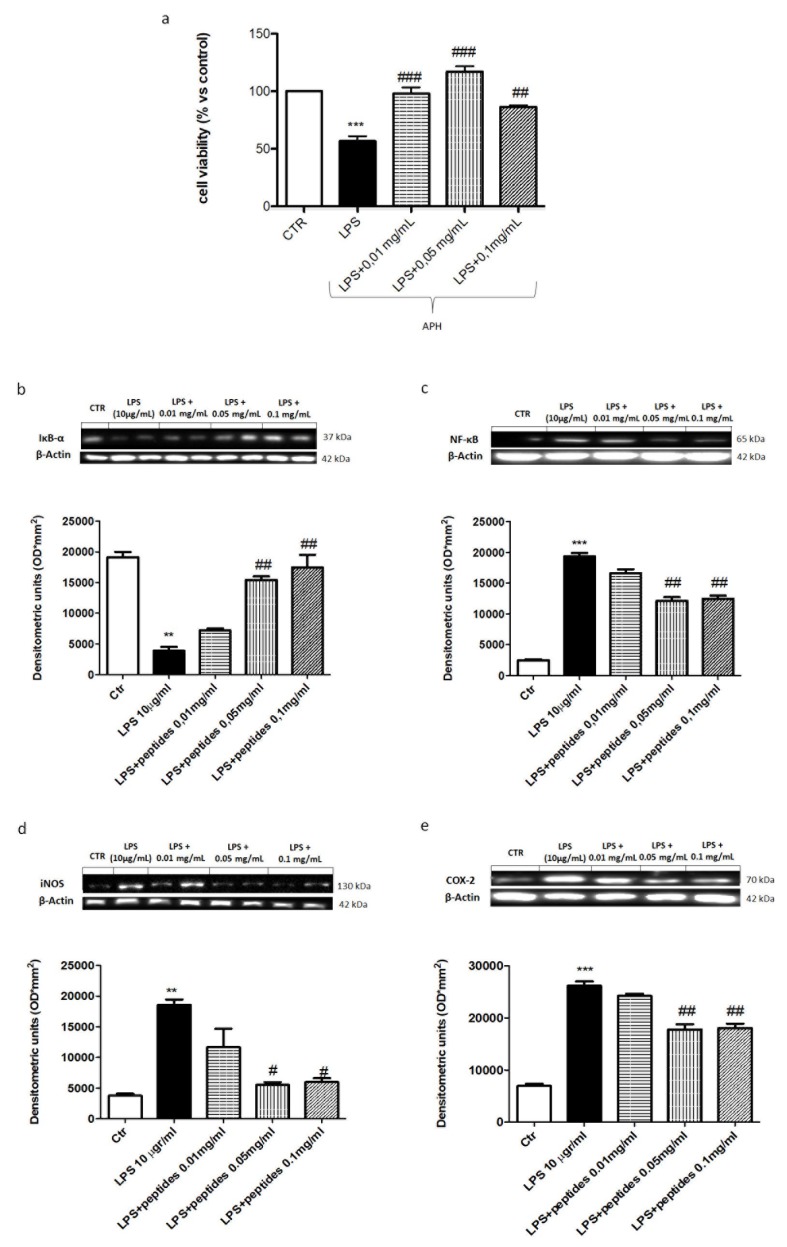
Effect of APH on inflammatory pathway on macrophages RAW 264.7. The incubation of cells with LPS significantly reduced cell viability compared to the control group, whereas pretreatment with APH at the concentrations of 0.01, 0.05 and 0.1 mg/mL significantly limited the reduction of cell viability (**a**). Western blot analysis for Ikb-α and NFκb demonstrated a significant decreased of Ikb-α and an increasing of NFκb following LPS stimulation (**b**,**c**, respectively). The pre-treatments with 0.05 and 0.1 mg/mL significantly decreased the cytosolic degradation of Ikb-α and reduced the nuclear traslocation of NFκb, respectively (**b**,**c**). The levels of iNOS and Cox2 were also reduced with APH treatment at concentrations of 0.05 and 0.1 mg/mL, respectively (**d**,**e**). Data are representative of at least three independent experiments. # *p* < 0.05; ## *p* < 0.01; ### *p* < 0.001 vs. LPS; ** *p* < 0.01; *** *p* < 0.001 vs. Ctr.

**Figure 3:**
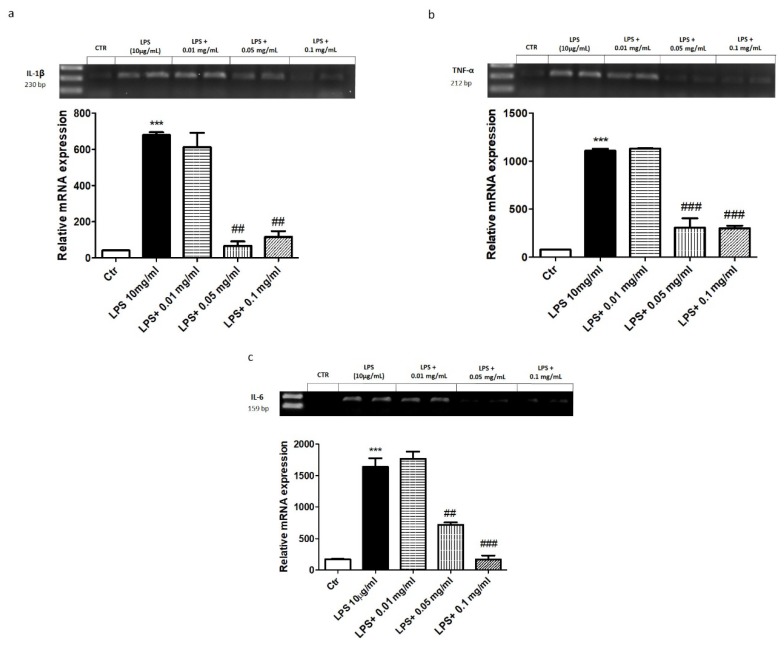
Effect of APH on cytokines mRNA expression on macrophages RAW 264.7. The expression of the pro-inflammatory cytokines *TNF-*α, *IL-1β* and *IL-6* were evaluated by RT-PCR (**a**,**b**,**c**). A significant decrease in *IL-1β* (230 bp), *TNF-α* (212 bp) and *IL-6* (159 bp) mRNA expression following LPS stimulation was evident. Moreover, the concentrations of 0.05 and 0.1 mg/mL decreased cytokine mRNA levels significantly. Data are representative of at least three independent experiments. ## *p* < 0.01; ### *p* < 0.001 vs. LPS; *** *p* < 0.01 vs. Ctr.

To assess the anti-inflammatory activity of hydrolysates following LPS stimulation, we evaluated the NF-κB pathway by Western blot analysis. The cells stimulated with LPS (10 μg/mL for 24 h) considerably increased NF-κB expression compared to the control group (Figure 2). The treatment with hydrolysates at concentrations of 0.05 and 0.1 mg/mL significantly reduced NF-κB nuclear translocation more efficiently than the treatment with APH at 0.01 mg/mL. Moreover, the involvement of NF-κB was confirmed by IκB-α degradation that was markedly prevented by hydrolysates at 0.05 and 0.1 mg/mL following LPS stimulation (Figure 2b,c). Moreover, we evaluated the effect of hydrolysates on pro-inflammatory enzymes such as COX-2 and iNOS following LPS stimulation by western blot analysis. A significant increase in COX-2 and iNOS expression was evident 24 h after LPS stimulation compared to the control group (Figure 2d). Instead, treatment with hydrolysates significantly decreased COX-2 and iNOS expression at the concentration 0.05 and 0.1 mg/mL. The concentration of APH 0.01mg/mL was not able to reduce pro-inflammatory enzymes expression (Figure 2d,e). To test whether hydrolysates modulate the levels of pro-inflammatory cytokines, we studied *IL-1β*, *TNF-α* and *IL-6* expression in cellular lysates by using semi-quantitative RT–PCR analysis. A significant decrease in *IL-1β* (230 bp), *TNF-α* (212 bp) and *IL-6* (159 bp) mRNA expression following LPS stimulation was evident. Moreover, the concentrations of 0.05 and 0.1 mg/mL decreased cytokine mRNA levels significantly (Figure 3a–c).

### 2.3. In Vitro Effect of APH on Oxidative Stress in Macrophages RAW 264.7

**Figure 4:**
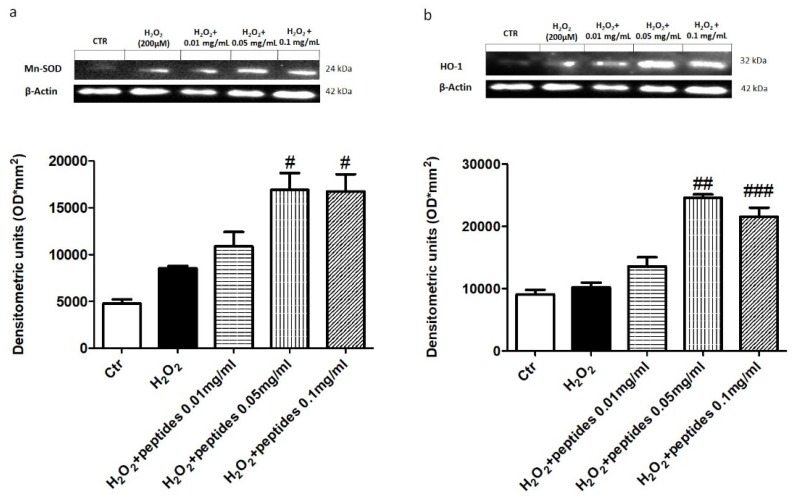
Effect of APH on anti-oxidant enzymes expression on macrophages RAW 264.7. The expression of the antioxidant enzymes MnSOD and HO-1 (**a**,**b**) was markedly decreased following H_2_O_2_ stimulation. The treatment with APH at the concentration of 0.05 and 0.1 mg/mL up-regulated MnSOD and HO-1 expression. Data are representative of at least three independent experiments. # *p* < 0.05; ## *p* < 0.01; ### *p* < 0.001 vs. H_2_O_2_.

**Figure 5:**
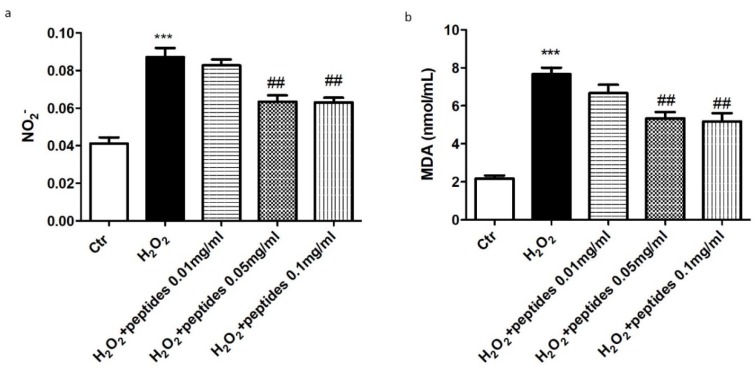
Effect of APH on nitrosative stress on macrophages RAW 264.7. The pre-treatment with APH at 0.05 and 0.1 mg/mL considerably reduced total NO levels after stimulation with H_2_O_2_ for 10 min (**a**). MDA levels were significantly increased in the cell lysates following H_2_O_2_ stimulation. Lipid peroxidation was significantly attenuated by the pre-treatment with APH at 0.05 and 0.1 mg/mL (**b**). Data are representative of at least three independent experiments. ## *p* < 0.01 vs. H_2_O_2_; *** *p* < 0.001 vs. Ctr.

To evaluate the effect of hydrolysates on oxidative stress, we evaluated the expression of the antioxidant enzymes MnSOD and HO-1 by western blot analysis. A marked decrease in both MnSOD and HO-1 expression was evident following H_2_O_2_ stimulation (Figure 4). Interestingly, treatment with hydrolysates at 0.05 and 0.1 mg/mL up-regulated MnSOD and HO-1 expression comparable to control group. As a marker of oxidative response, we investigated the total nitric oxide (NO) production in the supernatants of cells. Notably, 10 min following H_2_O_2_ stimulation, total NO levels were significantly higher compared to the control group. The pre-treatment with hydrolysates at 0.05 and 0.1 mg/mL considerably reduced total NO levels (Figure 5a). Moreover, we investigated the effect of hydrolysates on lipid peroxidation by MDA assay. Following H_2_O_2_ stimulation, MDA levels in the cell lysates were significantly increased. Lipid peroxidation was significantly attenuated by the pre-treatment with APH at 0.05 and 0.1 mg/mL (Figure 5b).

### 2.4. In Vivo Effect of APH on Atherosclerosis Markers in ApoE KO Mice

**Figure 6:**
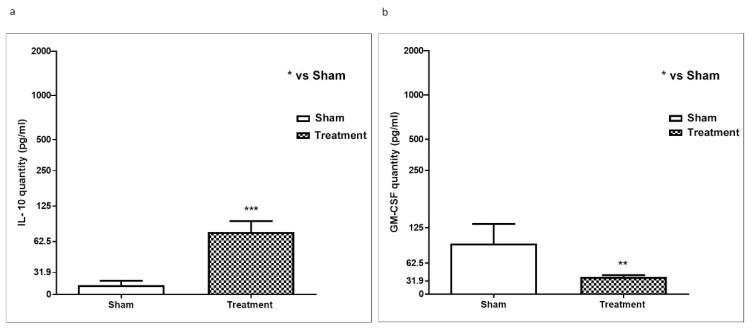
Effect of APH on IL-10 and GM-CSF expressionin ApoE KO mice. The levels of GM-CSF and IL-10 were evaluated by ELISA kit on plasma collected via retro-orbital blood samples from mice ApoE KO. The levels of GM-CSF were significantly decreased following the treatment with APH in ApoE KO mice (**a**). Moreover, ApoE KO mice showed a significant increasing in IL-10 following APH treatment (**b**). Data are representative of at least three independent experiments. **p < 0.01; *** *p*<0.001 vs. Sham.

Granulocyte macrophage colony-stimulating factor (GM-CSF, Csf2) is a growth factor for myeloid-lineage cells that has been implicated in the pathogenesis of atherosclerosis and other chronic inflammatory diseases. Thus, we evaluate by Elisa kit the level of GM-CSF on plasma collected via retro-orbital blood samples from mice ApoE KO. Our results showed a significant decrease in GM-CSF levels following the treatment with hydrolysates in ApoE KO mice (Figure 6a). Interleukin (IL)-10 in macrophages and T cells is involved in the modulation of several cellular processes that may interfere with the development and stability of the atherosclerotic plaque. Our data showed a substantial increasing in IL-10 in ApoE KO mice following hydrolysates treatment, demonstrating its protective role (Figure 6b). 

### 2.5. In Vivo Effect of APH on Inflammatory Pathway of ApoE KO Mice

**Figure 7:**
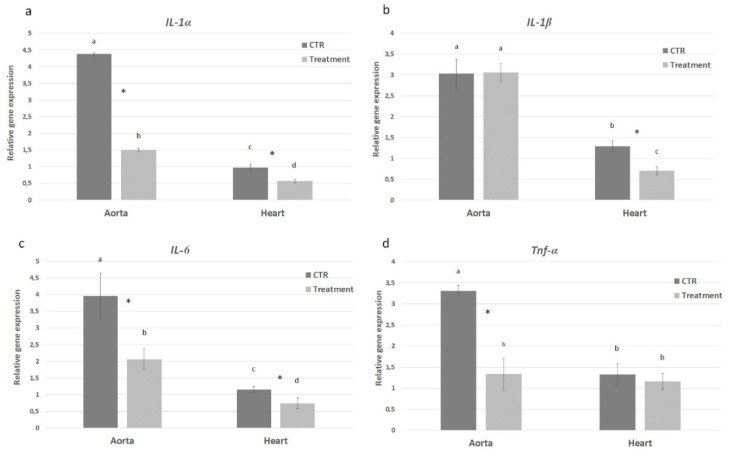
Effect of anchovy protein hydrolysates on proinflammatory cytokines. Quantitative gene expression of *IL-1α* (**a**), *IL-1β* (**b**), *IL-6* (**c**), and *Tnf-α* (**d**). Bars represent the mean ± SD (n = 6). Asterisks denote statistically significant differences with respect to control. Different letters indicate significant differences among groups (* *p* < 0.05).

**Figure 8:**
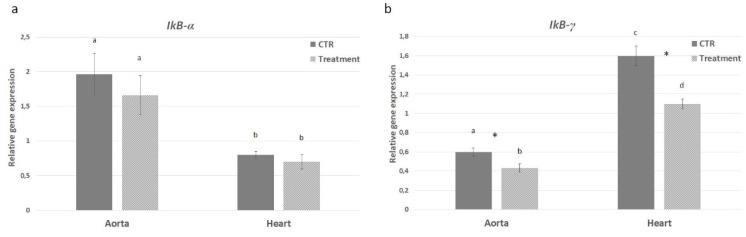
Effect of anchovy protein hydrolysates on NF-κB inhibitory proteins. Quantitative gene expression of *IKB-α* (**a**) and *IKB-γ* (**b**). Bars represent the mean ± SD (n = 6). Asterisks denote statistically significant differences with respect to control. Different letters indicate significant differences among groups (* *p* < 0.05).

**Figure 9:**
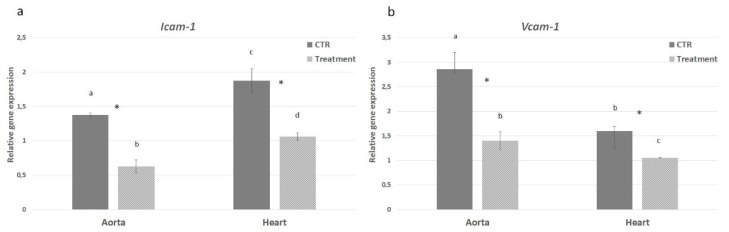
Effect of anchovy protein hydrolysates on expression of adhesion molecule. Quantitative gene expression of *Icam* (**a**) and *Vcam* (**b**). Bars represent the mean ± SD (n = 6). Asterisks denote statistically significant differences with respect to control. Different letters indicate significant differences among groups (* *p* < 0.05).

The levels of *IL-1α*, *IL-1β* and *IL-6* gene expression in heart and aorta tissues were evaluated from both control and treatment groups in order to assess the effect of hydrolysates on the inflammatory pathway. In the PH-treated mice a significant decrease of *IL-1α* mRNA level was observed in aorta (2.9 times) with respect to control group. A decreased difference, but still significant, was observed in heart tissue (1.7 times) (Figure 7a). On the contrary, the *IL-1β* gene expression was comparable in aorta from both experimental groups; in the heart, instead, *IL-1β* was down-regulated in the treated group with a difference of 1.8 times (Figure 7b). The mRNA level of *IL-6* showed a pattern of expression similar to *IL-1α* with significantly decreased expression in heart (1.54 times) and aorta (1.98 times) from APH-treated group (Figure 7c). Moreover, the NFk-B pathway was investigated to evaluate its possible involvement in the anti-inflammatory response exerted by the anchovy protein hydrolysates. *TNF-α* gene expression was down-regulated in aorta from treated mice (2.5 times); no difference in heart tissue was observed between experimental groups (Figure 7d). *IKB-α* gene expression was comparable in aorta and heart between control and treatment groups (Figure 8a). On the contrary the *IKB-γ* gene expression was down-regulated in both aorta and heart from treated mice (Figure 8b). Inflammatory markers *Icam* and *Vcam* were also investigated showing a decrease in mRNA level in both aorta and heart tissues from APH-treated mice, thus suggesting that anchovy protein hydrolysates exert an anti-inflammatory activity in ApoE KO mice (Figure 9).

### 2.6. In Vivo Effect of APH on Oxidative Stress of ApoE KO Mice

**Figure 10:**
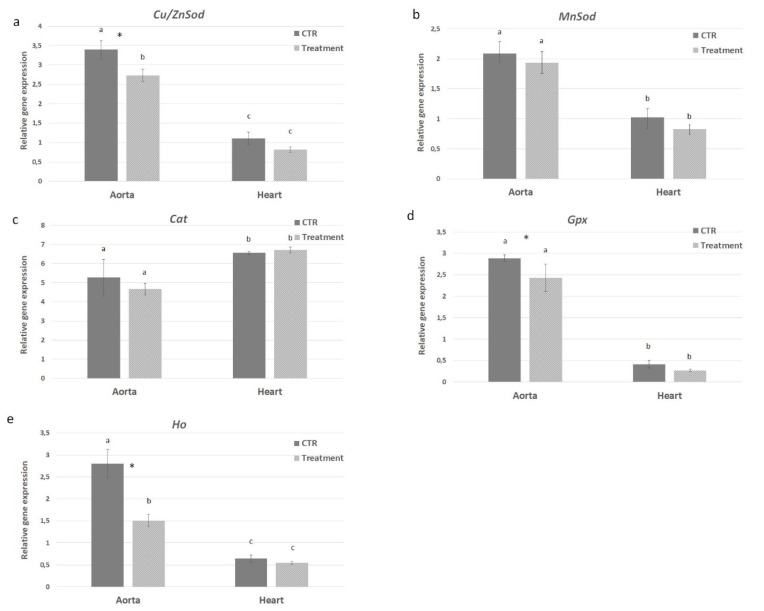
Effect of anchovy protein hydrolysates on oxidative stress response. Quantitative gene expression of *Cu/ZnSod* (**a**), *MnSod* (**b**), *Gpx* (**c**) and *Ho* (**d**). Bars represent the mean ± SD (n = 6). Asterisks denote statistically significant differences with respect to control. Different letters indicate significant differences among groups (* *p* < 0.05).

*Cu/ZnSod*, *MnSod*, *Gpx*, *Cat* and *Ho* mRNA expression levels were evaluated in both heart and aorta from control and APH-treated mice to assess the possible antioxidative role of anchovy protein hydrolysates. Gene expression analysis revealed that most of the antioxidative markers were modulated by the supplementation of protein hydrolysates in the diet, although at different levels (Figure 10). Only *Cat* expression did not show differences in heart and aorta from both experimental groups. Although mRNA expression level of *Cu/ZnSod*, *MnSod*, *Gpx* and *Ho* decreased in heart compared to aorta tissue, the treatment with protein hydrolysates induced a down regulation of superoxide dismutases: *Cu/ZnSod* expression was significantly decreased in aorta (1.24 times) from treated mice (Figure 10a); *MnSod* expression decreased 1.1 fold in aorta and 1.24 in heart from treated mice although this difference was not significant (Figure 10b). Similar expression patterns for *Gpx* and *Ho* mRNA levels were observed, with a significant difference in aorta *Ho* and *Gpx* levels where the treatment with protein hydrolysates induced a 1.87- and 1.19-times decrement in the gene expression, respectively (Figure 10c–e). The obtained results show that protein hydrolysates from anchovy are able to modulate the oxidative response and play an antioxidative role in ApoE KO mice.

## 3. Discussion

Marine organisms are a valuable source of bioactive proteins and peptides that can act as potential nutraceuticals, improving human health and preventing diseases through antihypertensive, anticoagulant, antioxidant and antiproliferative activities [21], as well as immunomodulating and lipid-lowering effects [18]. In the whole GFCM (General Fisheries Commission for the Mediterranean)*,* European anchovy *Engraulis encrasicolus* is one of the main commercial species landed, reaching more the 254,000 tonnes in the year 2016, with a 22% contribution (2014–2016 average) of the total landings and a 12% of the total landing value (FAO, 2018). Fish by-products have been receiving greater attention because of the possibility of obtaining high-value peptides that possess an excellent nutritional value. Protein hydrolysates obtained from *E. encrasicolus* waste, as used in this study, have been previously demonstrated to contain essential amino acids of high nutritional value. However, to the best of our knowledge, no studies have evaluated the potential biological activities of hydrolysates from *E. encrasicolus* in the prospective of their use as ingredient for functional food. The current investigation assessed the biological effects of protein hydrolysates obtained from anchovy (*Engraulis encrasicolus*) waste, focusing on the anti-oxidant and anti-inflammatory potential in both in vitro and in vivo models. In the in vitro model, murine macrophage RAW 264.7 cells were stimulated with lipopolysaccharide (LPS) from *Escherichia coli* or with H_2_O_2_ and then treated with protein hydrolysates at different concentrations [22]. The protein expression analysis of the inflammatory pathway showed that the treatment with hydrolysates exerted significant protection against LPS-induced inflammation, decreasing the expression of pro-inflammatory mediators, such as COX-2, preventing the degradation of IκB-α and inhibiting the nuclear translocation of NF-κB. Moreover, protein hydrolysates significantly decreased iNOS expression following LPS stimulation and were able to induce an activation of MnSOD and HO-1, decreasing the severity of oxidative stress. Therefore, in vitro results suggest that the protein hydrolysates from *Engraulis encrasicolus* may exert their protective effects through oxidative stress reduction by modulation of MnSOD and HO-1 and reducing pro-inflammatory mediators through NF-κB pathway. The in vivo effects of APH on inflammatory pathways were evaluated in aorta and heart of apolipoprotein E-knockout (C57BL/6) mice by quantitative gene expression. Results highlighted that the supplementation of protein hydrolysates in the diet was able to modulate the inflammatory response in apoE KO mice. Overall most of the markers analyzed (i.e., *IL-1α*, *IL-1β*, *IL-6*) showed a significant down regulation in both aorta and heart tissues of treated mice compared to control, thus indicating that APH in the diet can exert a beneficial effect, reducing the inflammatory parameters. Our results are in agreement with previous studies where the treatment with salmon protein hydrolysate in ApoE-deficient mice determined a decrease in interleukin levels in the plasma of treated mice [18]. A decrease in the expression of the adhesion molecule *Icam1*, as well as a reduction in *Vcam1*, was detected in both aortic and heart tissues of APH-treated mice, suggesting a local anti-inflammatory activity that could also reflect an anti-atherosclerotic effect of the APH-diet. During plaque development, indeed, accumulation of adhesion molecules contributes to foam cell formation [18]. Therefore, this study gives an indication that the anchovy by-product administration may have a protective role and could prevent atherosclerotic development through mechanisms linked to inhibition of inflammation. Anchovy protein hydrolysates tested in this study have been demonstrated to contain a high content of essential amino acids (42.0%), and 38% overall of proline, leucine, alanine, and aromatic amino acids [4]. The presence of these idrophobic and/or aromatic amino acids in the protein hydrolysates has been proved to play a scavenger role against dangerous molecules such as free radicals [23], and therefore an antioxidant effect. In the present study, we evaluated the transcriptional levels of a set of oxidative stress-related genes in both experimental and control mice. Our results showed a modulation of the antioxidant response by the supplementation of APH in the diet. Interestingly, the oxidative stress genes (*Cu/ZnSod, Gpx* and *Ho*) showed higher expression levels in transgenic mice respect to mice treated with protein hydrolysates in aorta tissue demonstrating that anchovy protein hydrolysates potentially exert antioxidant activities by reducing markers for oxidative stress. Moreover, the gene expression data showed a tissue-specific gene expression level highlighting a greater involvement of aorta in the protective role of APH against the oxidative stress. In heart tissue, indeed, the gene expression of *Cu/ZnSod*, *MnSod*, *Cat*, *Gpx* and *Ho* were statistically comparable between treated and control groups, suggesting that protein hydrolysates did not affect the antioxidant defence system in the heart of apoE^-/-^ mice. These results are in accordance with previous findings by Parolini et al. [18] that found the antioxidant response in heart of apoE^-/-^ mice fed with salmon protein hydrolysates to be unchanged. Overall, results from oxidative stress gene expression, highlighting a reduction of oxidative stress biomarkers, strongly suggest the APH antioxidant activity. Furthermore, the decrease in GM-CSF levels together with a substantial increase of IL-10 in ApoE KO mice following hydrolysate treatment demonstrates their protective role, thus supporting the use of protein hydrolysates as anti-inflammatory and antioxidant agents. Despite of the increasing interest in food derived bioactive peptides and the documented physiological functions, the molecular mechanisms underlying their biological activities thus far remain to be elucidated. The interactions of these protein hydrolysates and small bioactive peptides with proteins/receptors in vitro and their modifications during gastrointestinal digestion and absorption have been complicated the efforts to identify the specific metabolite(s) responsible for the observed physiological effects in different models. Multidisciplinary approaches could contribute to understanding the beneficial effects of these bioactive peptides in the perspective of using them as functional food [24]. Overall, the findings from both the in vitro and in vivo models showed that protein hydrolysates obtained from anchovy waste can modulate the inflammatory and oxidative stress biomarkers indicating that APH exert anti-inflammatory and antioxidant activities. The nutritional content of APH, together with their biological activities herein reported, highlight the possibility of obtaining high-value products from fish waste and to use these bioactive protein hydrolysates from *Engraulis encrasicolus* as potential nutraceuticals in food and pharmaceutical industries in the next future.

## 4. Materials and Methods 

### 4.1. Hydrolysate Preparation

The anchovy protein hydrolysate was produced by enzymatic hydrolysis from anchovy by-products (viscera) as detailed by Mangano et al. [4]. Briefly, frozen anchovies were ground into mince and, after pH adjustment, the commercial proteases (1.1:1.0:0.9 for Protamex: Flavourzyme 500 mg: Alcalase 2.4 L, Novozymes China Inc., Guangzhou, China) were added. The mixtures were incubated in a 5 L bioreactor (BIOSTATV R B Sartorius, Italy) in batch mode under strictly controlled parameters (pH 7.5, temperature 50 °C and agitation 150 rpm). To avoid nonenzymatic mechanisms all the parameters were constantly monitored. Hydrolysis was carried out for 3 h and stopped by raising the temperature to 90 °C for 15 min. The inactivated homogenates were filtered and after centrifugation (8000 rpm for 15 min at 4 °C), the collected supernatant was dehydrated in a Mini Spray Dryer B-290 (Buchi Italia s.r.l.) with a vanned wheel rotating at high speed and a concurrent drying chamber. Through a peristaltic pump, the solutions were fed into the chamber at a constant flow rate (15 mL/ min) under controlled temperature. The dried powders were collected in a single cyclone air separator system and subsequently used to determine the protein content by the 938.08 AOAC methodology (AOAC, 1993). High-performance liquid chromatography was used to assess the amino acid composition of the protein hydrolysates (Table 1).

### 4.2. In Vitro Studies on RAW 264.7

Murine RAW 264.7 macrophage cell line was obtained from American Type Culture Collection (ATCC) and cultured in DMEM supplemented with 10% FBS, 100 units/mL penicillin, 100 µg/mL streptomycin and maintained in a Thermo carbon dioxide incubator (Thermo Fisher Scientific, Waltham, MA, USA) at 37 °C with a humidified atmosphere of 95% air and 5% CO_2_. For inflammatory study, cells were incubated with 10 μg/mL of LPS for 24 h and treated with hydrolysates for 24 h; meanwhile, to simulate oxidative stress, cells were pre-treated with hydrolysates at different concentrations and then were stimulated with H_2_O_2_ at the final concentration of 200 μM for 10 min.

#### 4.2.1. Cell Viability Assay (MTT Assay)

The cellular viability of RAW 264.7 cell line was assessed using a mitochondria-dependent dye for live cells (tetrazolium dye; MTT) to formazan. Cultures were pre-treated with increasing concentrations (0.01–100 mg/mL) of the test compound for 24 h and then incubated at 37 °C with MTT (0.2 mg/mL) for 1 h. The medium was removed and the cells lysed with dimethyl sulfoxide (DMSO) (100 μL), as previously described by Campolo et al. [25]. The extent of reduction in MTT to formazan was quantified by measurement of optical density at 550 nm with a microplate reader.

#### 4.2.2. Western Blot Analysis

RAW 264.7 cells were washed twice with ice-cold phosphate buffered saline (PBS), harvested and resuspended in Tris-HCl 20 mM pH 7.5, NaF 10 mM, 150 μL NaCl, 1% Nonidet P-40 and protease inhibitor cocktail (Roche). Cell lysates were centrifuged and protein concentration was estimated by the Bio-Rad protein assay using bovine serum albumin as standard. Samples were heated at 95 °C for 5 min, and the same amounts of protein separated on 12% SDS-PAGE gel and then blotted to a PVDF membrane (Immobilon-P). The membrane was incubated overnight at 4 °C with anti-Manganese SOD (MnSOD) (1:500, Millipore); anti-Heme Oxigenase (HO-1) (1:500, Santa-Cruz Biotechnology, Dallas, Texas, USA); anti-NF-κB (1:500, Santa-Cruz Biotechnology); anti-IκB-α (1:500, Santa-Cruz Biotechnology), anti-COX-2 (1:500; Cayman) and iNOS (1:500, Santa Cruz). Relative expression of bands were detected respectively: for HO-1 (approximately 32 kDa), MnSOD (approximately 24 kDa), IκB-α (approximately 37 kDa), NF-κB (approximately 65 kDa), COX-2 (approximately 70 kDa), and iNOS (approximately 130 kDa); moreover, to ascertain that blots were loaded with equal amounts of protein lysate, they were also incubated with the antibody β-actin (1:500; Santa Cruz Biotechnology). Signals were detected with enhanced chemiluminescence (ECL) detection system reagent according to the manufacturer’s instructions (Thermo, USA). The relative expression of the protein bands was quantified by densitometry with BIORAD ChemiDocTMXRS+software and standardized to β-actin and lamin A/C levels.

#### 4.2.3. Determination of Malondialdehyde (MDA) Levels o TBARS

RAW 264.7 cells (1 × 10^5^ cells/well) were seeded in poly l-lysine-coated six-well plates. The cells were harvested to detect the levels of malondialdehyde (MDA) using the MDA assay kit.

#### 4.2.4. RT-PCR 

Total RNA (2 μg) was extracted from RAW 264.7 (4.5 × 10^5^ cells) and reverse transcribed cDNA was used as a template for RT-PCR in a T100 Thermal Cycler (Bio-Rad Hercules, California, USA) with Taq polymerase (Life Technologies). cDNAs underwent 30 cycles for *TNF-α*, *IL-6* and *IL-1β*, each one performed at 94 °C for 1 min, melting temperature (Tm) 55 °C for 45 sec and 72 °C for 55 sec. Amplified products were separated by 2% agarose gel electrophoresis. Fragments of DNA were detected under ultraviolet light. The primer sets shown in Table 2 were used to detect specific PCR products.

### 4.3. In Vivo Experimental Design

The Animal Welfare Organization (OPBA) approved the study (authorization number: 771/2018-PR). The study was conducted according to Italian regulations on use of animals for experiment (D.M.116192), as well as to EEC regulations (O.J. of E.C. L 358/1 12/18/1986) for animal care. The animals were kept at Experimental Zooprophylactic Institute of Sicily “A. Mirri”, Palermo, Italy (code 28875, Ministerial authorization 14/2015-UT). Thirty female six-month-old B6.129P2-ApoE^−/−^ mice from the breeding strain C57BL/6 were kept under standard laboratory conditions (12-h light cycle, temperature 22 ± 2 °C, humidity 55 ± 5%), with free access to food and water ad libitum. After 1 week of acclimatization under these conditions, mice were randomly divided into two groups—control and anchovy protein hydrolysates (APH) treatment—of 15 mice, in 3 cages per group with a density of 5 mice per cage. Although apoE^-/-^ mice spontaneously develop atherosclerosis, both groups (control and treatment) were fed a high-fat diet (21% w/w) to accelerate the atherosclerotic formation. In the treatment diet, 10% w/w casein was replaced with an equal amount of APH. The anchovy protein hydrolysate was produced by enzymatic hydrolysis from anchovy by-products (viscera) as detailed by Mangano et al. [4]. After 12 weeks (84 days), mice from control and treatment diets were dissected, and harvested tissues (heart and aorta) were subsequently stored in RNA (Invitrogen).

#### 4.3.1. ELISA Test on Plasma from ApoE KO Mice

According to the manufacturer’s instructions, a sandwich enzyme-linked immunosorbent assay (ELISA) method with mAbs recognizing GM-CSF and IL-10 was used. The levels of cytokines released on plasma, collected via retro-orbital blood sample from mice knock-out ApoE, were calculated based on absorbance values measured at 450 nm with a microplate reader and standard curves that were generated with recombinant cytokines. The results were expressed in picograms per milliliter.

#### 4.3.2. RNA Extraction and cDNA Synthesis

Total RNA was isolated from heart and aorta tissues from control and treated mice. RNA extraction was performed using TRIsure reagent (Bioline, France) and TissueLyser LT (QIAGEN Srl, Italy) following the manufacturer’s recommendations. RNA quality and quantity were assessed by 1% (w/v) agarose gel electrophoresis and Nanodrop spectrophotometer (Thermo Fisher Scientific, Italy). To remove any potential genomic DNA contamination, cDNA synthesis was performed after gDNA wipeout buffer treatment using the QuantiTect reverse transcription kit (Qiagen). 1μg total RNA for all the tissues from control and APH-treated mice was reverse transcribed according to manufacturer’s instructions. 

#### 4.3.3. Real-Time PCR (qPCR)

The mRNA level of oxidative stress- and inflammatory-related genes were quantified by real-time PCR using SYBR Green chemistry (Qiagen) in a Rotor-Gene Q 2plex Hrm thermocycler (Qiagen) as detailed by Giannetto et al. [26]. cDNA samples diluted 25-fold were run in duplicate, and in each reaction, no template or minus reverse transcriptase controls were included. A five-point standard curve of a 5-fold dilution series (1:1 to 1:32) from pooled RNA [27] was used to determine the PCR efficiency. The geNorm software (http://medgen.ugent.be/~jvdesomp/genorm/) was used to calculate the normalization factor from the two most stable reference genes, *Gadph* and *36b4*, to correct the raw target gene data. All sets of primers are listed in Table 2. PCR reactions were performed using the following parameters: 95 °C (15 min) followed by a two-step cycling of 95 °C (5 s) and combined annealing/extension at 60 °C (10 s) for 40 cycles. Melting curves analysis was used to confirm the specificity of each reaction. 

#### 4.3.4. Statistical Analysis

Analysis of variance followed by Student-Newman-Keuls post hoc tests was performed to assess differences in expression levels of the evaluated genes between control and treated mice using SigmaPlot (Systat software). Significance levels were set at P values of less than 0.05. For the in vitro study and the ELISA kit for IL-10 and GM-CSF, all values are expressed as mean ± standard error of the mean (SEM) of ‘n’ observations. The results were analyzed by one-way analysis of variance (ANOVA) followed by a Bonferroni posthoc test for multiple comparisons. A *p*-value of less than 0.05 was considered significant.

## Figures and Tables

**Table 1:** Amino acid composition of the anchovy protein hydrolysates analyzed by HPLC [4].

Amino Acids	Symbol	Amino Acids (g/100 g)
Isoleucine	ILE	3.26 ± 0.07
Leucine	LEU	6.87 ± 0.07
Lysine	LYS	10.94 ± 0.05
Methionine	MET	2.56 ± 0.02
Phenylalanine	PHE	6.83 ± 0.05
Threonine	THR	2.42 ± 0.02
Valine	VAL	4.19 ± 0.04
Arginine	ARG	8.83 ± 0.05
Glycine	GLY	10.87 ± 0.03
Proline	PRO	5.86 ± 0.05
Tyrosine	TYR	3.34 ± 0.02
Alanine	ALA	12.06 ± 0.04
Glutamic Acid	GLU	11.77 ± 0.09
Other		10.20
Total		100

**Table 2:** List of primer sets used in this study.

Primers	Sequence (5′ – 3′)	E (%)
**qPCR**		
*IL-1α_F*	CCTACTCGTCGGGAGGAGAC	99
*IL-1α_R*	GCAACTCCTTCAGCAACACG	
*IL-1β_F*	TCGCAGCAGCACATCAACA	90
*IL-1β_R*	GGTCCACGGGAAAGACACAG	
*IL-6_F*	GCCTTCTTGGGACTGATGCT	92
*IL-6_R*	CATTTCCACGATTTCCCAGAG	
*TNF-α_F*	CAACGGCATGGATCTCAAAG	95
*TNF-α_R*	CTTGACGGCAGAGAGGAGGT	
*IKB-α_F* ^a^	AGCATCTCCACTCCGTCCTG	109
*IKB-α_R* ^a^	CGTGGATAGAGGCTAGGTGC	
*IKB-γ_F* ^b^	AACAAGCACCCCTGGAAGAA	108
*IKB-γ_R* ^b^	ACAGCGTTCCCTCAGCATCT	
*Icam_F* ^c^	GAGTGGACCCAACTGGAAGC	97
*Icam_R* ^c^	CGGAAACGAATACACGGTGA	
*Vcam_F*	TGCTCAAATCGGTGACTCCAT	92
*Vcam_R*	GTGGGCTGTCTATCTGGGTTCT	
*Cu/ZnSod_F*	GAAGAGAGGCATGTTGGAGACC	102
*Cu/ZnSod_R*	TCTTGTTTCTCATGGACCACCA	
*MnSod_F*	ATCAAGCGTGACTTTGGGTCTT	95
*MnSod_R*	AAGCGACCTTGCTCCTTATTGA	
*Cat_F*	ACAGAGAGCGGATTCCTGAGAG	110
*Cat_R*	CTCTCCTCCTCGTTCAACACCT	
*Gpx_F*	GACACCAGAATGGCAAGAATGA	91
*Gpx_R*	TCTCACCATTCACTTCGCACTT	
*Ho_F*	GCACAGGGTGACAGAAGAGG	93
*Ho_R*	GTGAGGACCCACTGGAGGAG	
*Gadph_F*	TCCATGACAACTTTGGCATTG	98
*Gadph_R*	TCACGCCACAGCTTTCCA	
*36b4_F*	GGACCCGAGAAGACCTCCTT	99
*36b4_R*	GCACATCACTCAGAATTTCAATGG	
**Semiquantitative PCR**		**Product size**
*mm IL-6_F*	AGTTGCCTTCTTGGGACTGA	159 bp
*mm IL-6_R*	TCCACGATTTCCCAGAGAAC	
*mm IL-1β_F*	GCCCATCCTCTGTGACTCAT	230 bp
*mm IL-1β_R*	AGGCCACAGGTATTTTGTCG	
*mm TNF-α_F*	AGCCCCCAGTCTGTATCCTT	212 bp
*mm TNF-α_R*	CTCCCTTTGCAGAACTCAGG	

E, qPCR Efficiencies.
